# Visual Impairment and Cancer Risk: A Nationwide Cohort Study of Adult Swedish Men and Women

**DOI:** 10.3390/cancers18010147

**Published:** 2025-12-31

**Authors:** Leda Pistiolis, Henrik Litsne, Roger Olofsson Bagge, Kristian F. Axelsson

**Affiliations:** 1 Department of Surgery, Institute of Clinical Sciences, Sahlgrenska Academy, University of Gothenburg, 41390 Gothenburg, Sweden; 2 Department of Surgery, Sahlgrenska University Hospital, Region Västra Götaland, 41345 Gothenburg, Sweden; 3 Sahlgrenska Osteoporosis Center, Institute of Medicine, Sahlgrenska Academy, University of Gothenburg, 40530 Gothenburg, Sweden; 4 Region Västra Götaland, Närhälsan Norrmalm Health Center, 54940 Skövde, Sweden

**Keywords:** visual impairment, cancer risk, retrospective cohort study, health disparities

## Abstract

**Background:** Visual impairment is linked to a broad spectrum of health conditions. Nevertheless, there is an absence of large cohort studies investigating cancer risk in the visually impaired compared to seeing controls. The aim of this study was to investigate whether visually impaired adults have an increased risk for various types of cancer, compared to seeing controls. **Methods:** This was a retrospective cohort study comparing visually impaired adults aged 40 and over and 1:5 matched controls (by sex, birth year, and county of residence), between 2002 and 2018. Data were extracted from the Swedish National patient Registry, Statistics Sweden and from the Swedish Cause of Death Registry. A total of 48,493 adult Swedish men (44%) and women (56%), with a median age of 77 years (IQR: 64–86), with various degrees of visual impairment, from mild to total blindness, and 242,465 matched controls were followed up for a median of 5.1 (IQR 2.9–8.6) and 6.0 (IQR 3.6–9.8) years, respectively. The main outcomes included overall cancer incidence and incidence of different types of cancer. **Results:** Data analysis showed that visually impaired adults had an 18% significantly increased risk of any cancer (HR 1.18, 95% CI 1.16–1.20) when compared to seeing controls. The risk was also significantly increased for most cancers, with HRs ranging from 2.59 (95% CI 2.21–3.03) for brain cancer to 1.19 (95% CI 1.11–1.27) for breast cancer. No interaction was observed between cancer of any type and sex (*p* = 0.47). An elevated risk for all cancers in the visually impaired was observed across all age-groups: HR of 1.37 (95% CI 1.30–1.43) for ages 40–64, HR 1.17 (95% CI 1.13–1.20) for ages 65–79, and HR 1.11 (95% CI 1.08–1.15) for ages over 80. The risk of death was 60% higher among the cases compared to controls (HR 1.60, 95% CI 1.58–1.63). **Conclusions:** In this retrospective study of older adults, visual impairment was associated with increased cancer risk both overall and for specific types of cancer, in both sexes and across different age divisions of the cohort. These findings indicate the need for adjustments in health care polices in order to ensure equity in medical services.

## Simple Summary

This study aimed to investigate whether visually impaired adults have a higher cancer risk compared to sighted individuals. This was a retrospective cohort study comparing 48,493 visually impaired adults to their 242,465 matched controls. The findings demonstrate that visually impaired adults have a significant overall higher risk of cancer. The risk is also significantly elevated for most specific types of cancers examined. No difference was found between the sexes. An elevated cancer risk was observed for all age groups of the cohort. The findings underscore the necessity of targeted reforms in healthcare policies in order to ensure equitable distribution and access to health care services.

## 1. Introduction

Visual impairment (VI), ranging from mild forms to total blindness, represents a worldwide health problem with medical, social, psychological, and economic implications. In the adult population, leading causes include cataracts, uncorrected refractory error, glaucoma, age-related macular degeneration, and diabetic retinopathy [1]. Furthermore, there is geographical variation, with age-related macular degeneration being most prominent in high-income countries with an older population and uncorrected refractory errors and cataract being most prominent in lower- and middle-income countries, reflecting existing socioeconomic disparities [1,2]. The global burden of VI increased between 1990 and 2021, in spite of improvements in healthcare [3]. With ageing of the global population and the increase in diabetic retinopathy, the global burden is expected to rise [4].

However, the consequences of VI, affect more than just sight itself. A systematic review from 2021 showed that VI is associated with reduced quality of life [5]. Similarly, a cross-sectional analysis showed that visually impaired individuals, compared to controls, have more medical comorbidities, including cancer [6]. Between 1991 and 2009, a total of seven studies investigated the incidence of various types of cancer, in general, and of breast cancer in particular, in visually impaired/blind individuals or women, respectively [7,8,9,10,11,12,13]. Five came from the Scandinavian region [8,9,10,11,13] and two from the United States [7,12]. Five studies examined mainly breast cancer incidence/risk [7,10,11,12,13] and two also included other types of cancer [8,9]. In general, total blindness was found to confer an advantage for breast cancer and even prostate cancer risk, whereas it had no effect, or was even found to be unfavorable for, the risk for other cancers. A dose–response relationship was noted in some studies, with standardized incidence ratios for any type of cancer [8] and breast cancer [10,13] decreasing, with increasing severity of VI. All studies, except for one [12] compared cancer incidence/risk in the visually impaired to that of the general population of the corresponding country. Study populations ranged from 1392 [12] to 17,557 [9,13]. Even though studies on breast cancer seem to agree on a lower risk among the visually impaired, there is disagreement concerning the overall incidence of cancers.

The purpose of this study was to investigate the risk of various types of cancers in the visually impaired, comparing each one with five population controls, matched for age, sex, and region of residence. By establishing a more solid framework for the comparison of results, we drew safer conclusions, which can help inform health care policy decisions.

## 2. Materials and Methods

### 2.1. Study Design

This nationwide cohort study used national registries in Sweden to assess the risk of different cancers in patients with various degrees of VI. This study was approved by the Swedish Ethical Review Authority (Dnr: 2024–05343–01). The Strengthening the Reporting of Observational Studies in Epidemiology (STROBE) guidelines were followed [14].

### 2.2. Data Sources

Data regarding VI, cancer, and other comorbidities were retrieved from The National Patient Registry and included hospital-based diagnoses from inpatient [15] and outpatient visits [16]. Socioeconomic data were retrieved from Statistics Sweden and the date of death was retrieved from the Swedish Cause of Death Registry. In Sweden, all inhabitants are assigned a personal identification number at birth or at the time of immigration, enabling cross-reference between the different registries.

### 2.3. Study Population

This study included all Swedish men and women 40 years or older with varying degrees of VI (ICD-10 diagnoses starting with H54) between 2002 and 2018 and five population controls matched by sex, birth year, and county of residence. In Sweden, VI is assessed by an ophthalmologist. Coding is according to ICD-10, which classifies VI in four categories for bilateral and four for unilateral involvement. In bilateral cases, diagnosis and coding are based on visual acuity in the better eye (Table S1, Supplement). The date of the case patients’ first H54 diagnosis was used as a baseline for the case patient and the five corresponding controls. Control patients were required to lack a previous H54 diagnosis. Since ICD-10 was introduced in 1997, starting this study in 2002 ensured a minimum history of five years of prevalent cancer data for all patients.

### 2.4. Outcome Variables

The primary outcome was cancer incidence, based on ICD-10 diagnoses from the National Patient Registry. Secondary outcomes included the incidence of specific cancers: oral neoplasms, esophagus, stomach, small intestine, colon, rectum, liver, pancreas, lung, melanoma, skin, breast, uterus, ovary, urinary bladder, brain, thyroid, lymphoma/leukemia, and solid metastasis. The follow-up time for all outcomes was censored at the end of the study (31 December 2021), emigration, and death, and the controls were also censored at H54 diagnosis.

### 2.5. Baseline Variables

Baseline variables included patient age, sex, inclusion year, previous cancers, and Charlson comorbidity index (CCI) [17]. The latter was calculated using the following conditions and weights; dementia (1), ischemic heart diseases (1), congestive heart failure (1), cerebrovascular diseases (1), diseases of arteries, arterioles, and capillaries (1), chronic pulmonary disease (1), chronic liver disease (1), tumor without metastasis (2), lymphoma or leukemia (2), diabetes (1), diabetes with end organ damage (+1), renal failure (1), renal failure, moderate or severe (+1), hemiplegia (29), peptic ulcer disease (2), and metastatic solid tumor (6).

### 2.6. Methodological Considerations

Due to the diversity in both the etiology of VI and the risk factors pertaining to the different kinds of cancers, we chose not to adjust for more than just age, sex, and baseline year, and prevalence of the cancer in question, depending on cancer outcome. As a consequence, the associations found and presented are not independent of other underlying causes. However, this was deemed to be a fair trade off. The methodological approach used was independent of the type of cancer outcome, i.e., we first described crude incidence rates, then accounted for events at different time points during follow-up using Cox regression, and finally added adjustment for previous cancer of the same type as the outcome in question, thereby avoiding the need to exclude different patients depending on outcome and allowing the age–sex–country matching to remain intact. A subgroup analysis based on the different degrees of VI (ICD 10 H54) was also considered. However, H54 was divided in nine different groups based on both laterality and the severity of visual impairment (mild/moderate/severe/blind). The resulting groups were quite small (Table S1, Supplement) and would give rise to power concerns in case of subgroup analyses.

### 2.7. Statistical Analyses

Descriptive baseline statistics for the whole cohort are presented as counts with percentages for categorical variables and averages with standard deviations (SDs) for continuous variables. Event rates for all the outcomes were calculated as the number of events per 1000 person-years and are presented with exact Poisson 95% confidence intervals. Cox regression models were calculated for all the outcomes, with adjustment for age, sex, and baseline year, as well as with added adjustment for previous cancer (depending on the outcome). We performed subgroup analyses per sex and age groups. Statistical analyses were performed using R version 4.2.2 and R-Studio version 2023.03.0+386.

## 3. Results

### 3.1. Study Population

A total of 48,493 adults with VI aged 40 and over and 242,465 controls matched for age, sex, and inclusion year, were included in this study and followed for a median follow-up time of 5.8 (IQR 3.5–9.6) years. Their median age was 77 (IQR: 64–86) years. Fifty-six percent of them were women. The mean CCI was significantly higher in the VI group (1.48, SD 1.93) compared to controls (0.85, SD 1.49) (Table 1). The prevalence of any cancer during the last five years was significantly higher (10%) in the VI group compared to controls (7.8%). For specific cancer types, the prevalence was also higher in the visually impaired compared to controls and, in most cases, was also significant (Table 1).

### 3.2. Risk of Cancer in the Visually Impaired

The visually impaired patients were followed for a median time of 5.1 (IQR 2.9–8.6) years and their corresponding controls were followed for a median time of 6.0 (IQR 3.6–9.8) years. During follow-up, 11,087 visually impaired individuals (22.9%) and 54,697 matched controls (22.6%) were diagnosed with cancer of any type (C00-C97), translating to incidence rates of 43.1 (95% CI, 42.3–43.9) and 36.7 (95% CI, 36.4–37.0) per 1000 person-years, respectively. Compared to seeing controls, the visually impaired had an 18% increased risk of any cancer (HR 1.18, 95% CI 1.16–1.20), adjusted for age, sex, and baseline year, which remained unchanged when adjusting for previous cancer (HR 1.17, 95% CI 1.14–1.19).

**Figure 1:**
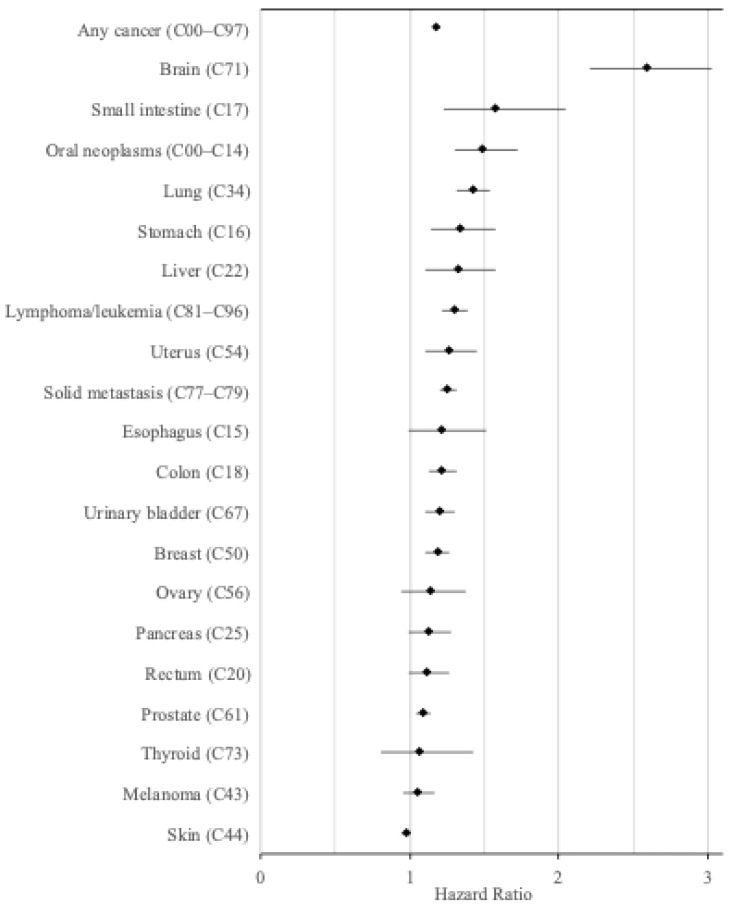
Hazard ratios for the incidence of various cancers in visually impaired individuals compared to an equal number of matched controls (adjusted for age, sex, and year).

The risk was significantly increased for most cancers, with the age, sex, and baseline year adjusted HR (95% CI) ranging from 2.59 (95% CI, 2.21–3.03) for brain cancer, 1.58 (1.23–2.05) for small intestine cancer, 1.49 (95% CI 1.30–1.72) for oral cancer, and 1.19 (95% CI 1.11–1.27) for breast cancer (Table 2, Figure 1). The HRs were not substantially changed after adjusting for previous cancer (Table 2).

### 3.3. Risk of Cancer per Sex

When adding an interaction term (sex x VI), in the age-, sex-, baseline-year-adjusted Cox model for any cancer, there were no significant interactions (*p* = 0.47). For the sex-specific cancers in the age-, sex-, and baseline-year-adjusted Cox, there was a 9% increase in prostate cancer in men with VI compared to seeing controls (Table S2, Supplement) and an 18%, 27%, and 14% increased risk of breast, uterus, and ovarian cancer in women with VI compared to seeing controls (Table S3, Supplement).

### 3.4. Risk of Cancer per Age Group

When adding an interaction term (sex x VI), in the age-, sex-, and baseline-year-adjusted Cox model for any cancer, there was a significant interaction (*p* < 0.001).

#### 3.4.1. Adult Population Between 40–64 Years

**Figure 2:**
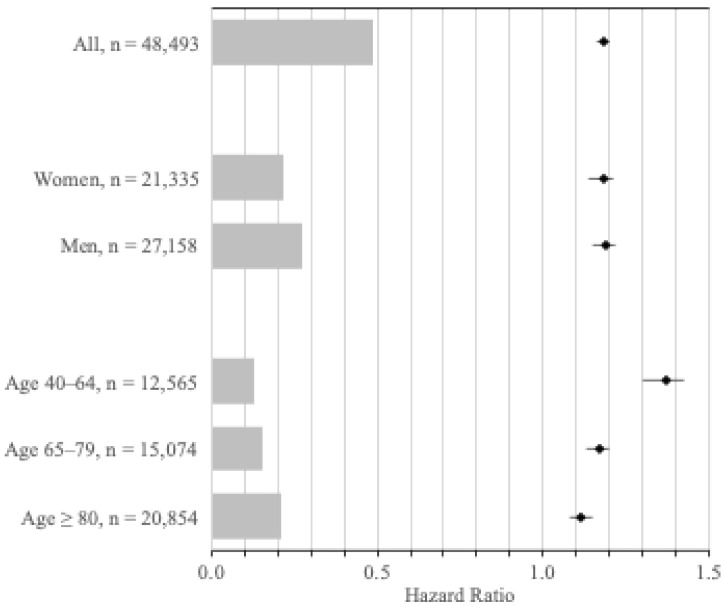
Hazard ratios for the incidence of any cancer, according to sex and age group. Hazard ratios for incident cancer (any type), adjusted for age, sex, and year, for visually impaired individuals for all patients, by sex, and by age group compared to an equal number of matched controls. The bars shaded in gray represent the number of visually impaired individuals at risk in each group.

The first subset of the cohort included 12,565 individuals with VI and 62,825 matched controls. During follow-up, 2168 visually impaired (17.3%) and 8847 matched controls (14.1%) were diagnosed with cancer of any type (C00-C97), translating to incidence rates of 20.8 (95% CI 19.9–21.7) and 15.4 (95% CI 15.1–15.8) per 1000 person-years, respectively. Compared to seeing controls, visually impaired patients had a 37% increased risk of any cancer (HR 1.37, 95% CI 1.30–1.43) (Figure 2), which remained unchanged when adjusting for previous cancer (HR 1.31, 95% CI 1.25–1.38) (Table S4, Supplement).

#### 3.4.2. Adult Population Between 65–79 Years

The second subset included 15,074 individuals with VI and 75,370 matched controls. During follow-up, 4359 visually impaired (28.9%) and 21,726 matched controls (28.8%) were diagnosed with cancer of any type (C00-C97), translating to incidence rates of 51.4 (95% CI 49.9–53.0) and 43.4 (95% CI 42.8–44.0) per 1000 person-years, respectively. Compared to seeing controls, visually impaired patients had a 17% increased risk of any cancer (HR 1.17, 95% CI 1.13–1.20) (Figure 2), which remained unchanged when adjusting for previous cancer (HR 1.16, 95% CI 1.13–1.20) (Table S5, Supplement).

#### 3.4.3. Adult Population Aged 80 Years and Older

The third subset included 20,854 individuals with VI and 104,270 matched controls. During follow-up, 4560 visually impaired patients (21.9%) and 24,124 matched controls (23.1%) were diagnosed with cancer of any type (C00-C97), translating to incidence rates of 66.9 (95% CI 65.0–68.9) and 58.0 (95% CI 57.2–58.7) per 1000 person-years, respectively. Compared to seeing controls, visually impaired patients had an 11% increased risk of any cancer (HR 1.11, 95% CI 1.08–1.15) (Figure 2), which remained unchanged when adjusting for previous cancer (HR 1.10, 95% CI 1.07–1.14) (Table S6, Supplement).

### 3.5. Mortality

During the median follow-up time of 5.8 (IQR 3.5–9.6) years, there were 26,307 deaths (54.2%) among the cases and 103,617 deaths (42.7%) among the controls, translating to incident rates of 89.1 (95% CI 88.0–90.1) and 61.0 (95% CI 60.7–61.4) deaths per 1000 person-years, respectively. In a Cox-regression model adjusted for age, sex, and baseline year, the risk of death was 60% higher in VI patients compared to seeing controls (HR 1.60 (95% CI 1.58–1.63)).

## 4. Discussion

The cohort consisted of all Swedish adults, aged 40 years and older, registered as visually impaired and their corresponding matched controls. The median age for both groups was 77 (IQR: 64–86) years, with age being a contributing factor to VI, cancer, and underlying diseases. The VI group had a higher CCI, suggesting that underlying diseases, such as diabetes, could account for both additional comorbidities and VI. This group also had a higher prevalence of any cancer in the previous five years. These findings suggest that VI may be linked to systemic health vulnerabilities, as a direct outcome or a contributing factor, rather than being a feature conferring protection against malignancy.

During the follow-up period, we found a statistically significant higher risk for developing cancer of any type in the visually impaired. Furthermore, there was a significantly higher risk for specific types of cancers, including brain, small intestine, oral neoplasms, lung, stomach, liver, lymphoma/leukemia, uterus, solid metastasis, colon, urinary bladder, and breast (in decreasing order of risk). Similar risk between the two groups was found for cancer of the esophagus, rectum, pancreas, melanoma, skin, ovary, thyroid, and prostate (the last being statistically significant). In comparison to their respective controls, visually impaired men had a higher risk for oral, rectal, pancreatic, and breast cancer, whereas visually impaired women had a higher risk for stomach and small intestinal cancer (Tables S2 and S3, Supplement). This could reflect biological differences, sex-specific risk-factor exposures, or even differences in health-seeking behavior. Among the different divisions of the cohort, there was a tendency for the incidence rate, in both visually impaired and controls, to increase with increasing age, as would be expected, whereas the hazard ratio for the visually impaired decreased. The adjusted hazard ratio decreased from 1.37 (ages 40–64) to 1.11 (ages 80 and older), indicating that additional parameters (age, most notably, but also pre-existing comorbidities) start becoming an important risk factor for cancer in both groups. This, is in accordance with the epidemiological observation that cancer, being a disease of decay, is more prevalent in the elderly [18,19], with age becoming the principle determining factor, potentially diluting the relative impact of other risk factors.

When comparing our results to those of the two previous studies investigating multiple cancers [8,9], our results are consistent with the Finnish study [9], depicting an increased overall cancer incidence in the visually impaired. For breast cancer, in particular, earlier studies have shown a decreased risk in the totally blind and generally a similar risk in the visually impaired. In contrast, our findings demonstrate a slightly increased risk in women and a moderately increased risk in men.

The overall tendency for increased cancer risk in the visually impaired could be partly explained by several confounding factors. The cohort consists of individuals aged 40 years and older. Age-related macular degeneration represents the most frequent cause of VI in Western societies [1] and age itself is also a risk factor for cancer. Diabetic retinopathy is another potential confounding factor. It affects patients with both type 1 and 2 diabetes and its incidence increases proportionally with age, disease duration, and suboptimal glycemic control [4]. Both abovementioned factors are expected to increase with the ageing world population. A recent study indicated an increased risk in cancer incidence in diabetics, which is further accentuated in patients with diabetic retinopathy [20]. Obesity, with or without the presence of diabetes, is another possible link between VI and cancer since it can, on its own, confer alterations in the visual system [21]. Along similar lines, the increased cancer risk found could be attributed to the more frequent medical visits of visually impaired individuals, resulting in more frequent physical examinations and diagnostic investigations, a phenomenon often described as the “surveillance effect”. On the other hand, there are conflicting data on this, with some studies referring to the “competing demands” hypothesis, which suggests that the presence of comorbidities might distract from the early symptoms of a tumor [22]. Ultimately, it would be difficult, if not impossible, to calculate the relative effect of either.

Of interest are our findings on brain cancer, which showed a marked increase in the visually impaired (HR, 2.59; 95%CI, 2.21–3.03), which is in accordance with the only study that examined brain cancer, showing similar results [9]. This could be due to several underlying causes: VI could be one of the primary symptoms of an undiagnosed brain cancer situated in the visual cortex or exerting pressure on the optic nerve [23]. Alternatively, the patient could be the carrier of a rare genetic condition, such as neurofibromatosis, which could be accountable for both [24,25].

Lifestyle factors could also be accountable. Smoking is one of the principle reversible causes of macular degeneration [26] and a risk factor for cancer. Physical activity and dietary habits among the visually impaired could also be implicated. Research has demonstrated that the visually impaired are less physically active and engage in more sedentary activities, with several failing to reach the recommended amount of physical activity [27,28,29]. Dietary issues could also play a role, since financial constraints and disability-related factors could impede the acquisition and preparation of nutritious food [9,30]. Last, but not least, marital status [31], age of parity, and age of menarche, shown in some studies to occur earlier in blind women [32], could also play a role.

In addition to the above, one should keep in mind that there is a reciprocal association between VI and socioeconomic status (SES). Lower SES has been found to be associated with a higher prevalence of VI due to variances in ophthalmological controls and therapy, including routine eye screening, refractive correction, access to cataract surgery, and management of chronic diseases such as glaucoma and diabetic retinopathy [33]. Furthermore, individuals of lower SES might come into contact with more risk factors affecting the integrity of their vision, with adverse consequences. Such factors include occupational hazards, environmental pollutants, and the higher prevalence of diabetes, other chronic diseases, and infectious diseases that increase the likelihood of developing visual problems [34,35,36]. On the other hand, VI itself can contribute to lower SES by reducing opportunities for higher education and access to higher-paying jobs, as well as career advancement [30,37,38]. The aforementioned parameters might impact an individual’s access to healthcare information, access to screening programs, and vaccinations, all of which could partly account for the higher incidence of cancer in visually impaired individuals [39,40]. Furthermore, in spite of their need for medical treatment and follow-up, some individuals with VI may have reduced access to medical care, primarily due to the associated costs, lack of insurance, or problems with transportation [41].

Previous studies investigating the risk of cancer and VI devoted a significant part of their discussions to the possible protective role of melatonin. The “melatonin hypothesis”, proposed by S. Stevens in 1987 [42,43], postulated that the suppression of melatonin production by light at night (LAN) or by exposure to electromagnetic radiation, can lead to an increased risk of breast cancer. His arguments were grounded on the suppressive effect of LAN, and electromagnetic radiation on melatonin production, and pre-clinical experimental evidence from that time pointing towards an inhibitory effect of melatonin on breast cancer. Since then, a substantial body of evidence has accumulated. Ophthalmological research has demonstrated that even in totally blind individuals, melatonin production can be inhibited after being exposed to bright light, indicating the transference of photic information from the retina to the hypothalamus [44,45,46]. This has to be taken into consideration when reviewing the results of a study demonstrating that blind women with no light perception have a lower risk of breast cancer compared to blind women with light perception [12]. At present, the true effect of VI on melatonin production is not known. Moreover, increasing evidence indicates variability in the magnitude of melatonin suppression by LAN [47,48,49]. Considering the aforementioned parameters in conjunction with our results, there is no relevance for the melatonin hypothesis as a possible explanation.

This study is, to the best of our knowledge, the largest to address the question of whether visually impaired adults have a different cancer incidence compared to seeing controls. Each individual in the VI group was matched to five controls, giving added strength to the statistical analysis. In order to maintain maximum power, all individuals included in this study were checked for cancer diagnosis five years before inclusion. Hence, instead of deleting all patients with previous cancers, we chose to adjust for any previous cancers of the same type under investigation. However, there are some limitations to this study. It contains individuals aged 40 years and older and could potentially miss cancers arising at a younger age (e.g., breast and lymphoma). Although the median follow-up period for the visually impaired was a mere 5.1 years, too short to develop some cancers, 10% of the visually impaired were followed for more than 13 and up to 20 years. An additional limitation is the lack of information pertaining to lifestyle factors, which would allow for necessary adjustments. Finally, being a retrospective registry study, it relies on previously recorded data and even though in Sweden the registries have nationwide coverage, they could be subject to missing data and miscoding, albeit to a small degree. Reviews of the Swedish Patient Registry concluded that it has, overall, good validity and little missing data [15,16].

As can be seen by the factors discussed above, the observed increased cancer incidence among the visually impaired can be explained as the cumulative effect of a combination of contributing mechanisms. Hereditary genetic diseases such as neurofibromatosis and xeroderma pigmentosum may predispose the individual to subsequent cancer development. Chronic conditions such as diabetes mellitus and auto-immune disorders, instigating a pro-inflammatory state, can contribute to both ocular pathology and cancer development. Lifestyle factors influenced by poor or absent eyesight, leading to reduced physical activity, a sub-optimal nutritious diet, obesity, and unhealthy habits such as tobacco and alcohol use, can increase susceptibility to carcinogenesis. Reduced accessibility to screening due to reduced access to health information and transportation issues can delay early diagnosis. SES factors can further aggravate the effect of lifestyle factors and contribute to lower education, occupation, and income. The individual impact of each of the above-mentioned parameters on the final outcome is difficult to estimate, depending itself on variables such as country of residence and the etiology of VI, to name a few.

Our findings call attention to several factors such as access to healthcare, screening, medical information and digital platforms that could pose a challenge for the visually impaired [39,50]. This is in accordance with the observed higher overall mortality among the visually impaired, suggesting that insufficient or restricted access to screening and healthcare services might lead to adverse health outcomes. Our results underscore the importance of targeted policy interventions and the need for a healthcare system that accommodates the needs of the patients and offers equity in the medical services provided.

## 5. Conclusions

Taken together, our findings agree, in part, with previous literature, showing that VI is associated with increased cancer risk overall, as well as for several major cancer types, in both sexes and across different age groups in the adult population. This calls for improvements in the provision of screening and health services to ensure access and equity for these patients. Further research is needed to identify underlying biological mechanisms, as well as the socioeconomic factors that could be involved, in order to better guide subsequent changes in healthcare policies and clinical practice.

## Figures and Tables

**Table 1:** Baseline characteristics of visually impaired individuals (H54) compared to population controls.

	H54	Ctrls	*p*-Value
	*n* = 48,493	*n* = 242,465	
**Age, median**	77 (IQR: 64–86)	77 (IQR: 64–86)	1.00
**Sex, *n* (%)**			1.00
**Men**	21,335 (44.0)	106,675 (44.0)	
**Women**	27,158 (56.0)	135,790 (56.0)	
**Baseline year, *n* (%)**			1.00
**2002–2006**	7911 (16.3)	39,555 (16.3)	
**2007–2010**	10,842 (22.4)	54,210 (22.4)	
**2011–2014**	13,399 (27.6)	66,995 (27.6)	
**2015–2018**	16,341 (33.7)	81,705 (33.7)	
**Charlson, mean ± SD**	1.48 ± 1.93	0.85 ± 1.49	<0.001
**0, *n* (%)**	21,219 (43.8)	153,496 (63.3)	
**1, *n* (%)**	8726 (18.0)	31,222 (12.9)	
**2, *n* (%)**	7959 (16.4)	31,694 (13.1)	
**≥3, *n* (%)**	10,589 (21.8)	26,053 (10.7)	
**Previous cancer last 5 years, *n* (%)**			
**Any cancer (C00–C97)**	4841 (10.0)	18,852 (7.8)	<0.001
**Oral neoplasms (C00–C14)**	137 (0.28)	344 (0.14)	<0.001
**Esophagus (C15)**	33 (0.07)	69 (0.03)	<0.001
**Stomach (C16)**	51 (0.11)	175 (0.07)	0.02
**Small intestine (C17)**	28 (0.06)	74 (0.03)	0.005
**Colon (C18)**	421 (0.87)	1417 (0.58)	<0.001
**Rectum (C20)**	200 (0.41)	649 (0.27)	<0.001
**Liver (C22)**	38 (0.08)	77 (0.03)	<0.001
**Pancreas (C25)**	56 (0.12)	128 (0.05)	<0.001
**Lung (C34)**	238 (0.49)	465 (0.19)	<0.001
**Melanoma (C43)**	275 (0.57)	1038 (0.43)	<0.001
**Skin (C44)**	1825 (3.8)	8745 (3.6)	0.10
**Breast (C50)**	579 (1.2)	2236 (0.92)	<0.001
**Uterus (C54)**	162 (0.33)	527 (0.22)	<0.001
**Ovary (C56)**	63 (0.13)	208 (0.09)	0.005
**Prostate (C61)**	811 (1.7)	3815 (1.6)	0.12
**Bladder (C67)**	272 (0.56)	1067 (0.44)	<0.001
**Brain (C71)**	113 (0.23)	66 (0.03)	<0.001
**Thyroid (C73)**	23 (0.05)	101 (0.04)	0.66
**Lymphoma/leukemia(C81–C96)**	393 (0.81)	1117 (0.46)	<0.001
**Solid metastasis (C77–C79)**	827 (1.7)	2031 (0.84)	<0.001

**Table 2:** Incident cancers in visually impaired individuals (H54) compared to population controls.

Incident Cancer Event	*n* (%)		Incident Rate per 1000 pys		HR (95%CI)	
	H54	Ctrls	H54	Ctrls	Adj. Age, Sex, Year	+ adj. Prev Cancer
**Any cancer (C00-C97)**	11,087 (22.9)	54,697 (22.6)	43.1 (42.3–43.9)	36.7 (36.4–37.0)	1.18 (1.16–1.20) **	1.17 (1.14–1.19) **
**Oral neoplasms (C00-C14)**	247 (0.51)	944 (0.39)	0.84 (0.74–0.95)	0.56 (0.52–0.59)	1.49 (1.30–1.72) **	1.41 (1.22–1.62) **
**Esophagus (C15)**	102 (0.21)	492 (0.20)	0.35 (0.28–0.42)	0.29 (0.26–0.32)	1.22 (0.99–1.51)	1.09 (0.88–1.35)
**Stomach (C16)**	173 (0.36)	760 (0.31)	0.59 (0.50–0.68)	0.45 (0.42–0.48)	1.34 (1.14–1.58) **	1.32 (1.12–1.56) **
**Small intestine (C17)**	75 (0.15)	269 (0.11)	0.25 (0.20–0.32)	0.16 (0.14–0.18)	1.58 (1.23–2.05) **	1.39 (1.08–1.81) *
**Colon (C18)**	767 (1.58)	3691 (1.52)	2.61 (2.43–2.81)	2.19 (2.12–2.26)	1.22 (1.13–1.32) **	1.20 (1.11–1.30) **
**Rectum (C20)**	306 (0.63)	1578 (0.65)	1.04 (0.93–1.16)	0.93 (0.89–0.98)	1.12 (0.99–1.27)	1.09 (0.97–1.23)
**Liver (C22)**	152 (0.31)	675 (0.28)	0.51 (0.44–0.60)	0.40 (0.37–0.43)	1.33 (1.11–1.58) *	1.28 (1.07–1.53) *
**Pancreas (C25)**	280 (0.58)	1467 (0.61)	0.95 (0.84–1.07)	0.86 (0.82–0.91)	1.13 (0.99–1.28)	1.12 (0.98–1.27)
**Lung (C34)**	782 (1.61)	3185 (1.31)	2.66 (2.47–2.85)	1.88 (1.82–1.95)	1.42 (1.32–1.54) **	1.37 (1.27–1.49) **
**Melanoma (C43)**	446 (0.92)	2477 (1.02)	1.52 (1.38–1.66)	1.47 (1.41–1.53)	1.05 (0.95–1.17)	1.03 (0.93–1.14)
**Skin (C44)**	3210 (6.62)	19,303 (7.96)	11.4 (11.0–11.8)	12.0 (11.8–12.1)	0.98 (0.94–1.01)	0.97 (0.94–1.01)
**Breast (C50)**	996 (2.05)	4692 (1.94)	3.42 (3.21–3.64)	2.80 (2.72–2.88)	1.19 (1.11–1.27) **	1.18 (1.10–1.27) **
**Uterus (C54)**	253 (0.52)	1115 (0.46)	0.86 (0.76–0.97)	0.66 (0.62–0.70)	1.27 (1.10–1.45) **	1.21 (1.05–1.38) *
**Ovary (C56)**	123 (0.25)	601 (0.25)	0.42 (0.35–0.50)	0.35 (0.33–0.38)	1.14 (0.94–1.38)	1.12 (0.92–1.36)
**Prostate (C61)**	2060 (4.25)	10,990 (4.53)	7.16 (6.85–7.47)	6.65 (6.53–6.78)	1.09 (1.04–1.14) **	1.11 (1.06–1.17) **
**Bladder (C67)**	701 (1.45)	3372 (1.39)	2.39 (2.22–2.58)	2.00 (1.93–2.07)	1.20 (1.11–1.30) **	1.21 (1.12–1.32) **
**Brain (C71)**	224 (0.46)	484 (0.20)	0.76 (0.66–0.87)	0.29 (0.26–0.31)	2.59 (2.21–3.03) **	1.91 (1.61–2.26) **
**Thyroid (C73)**	58 (0.12)	298 (0.12)	0.20 (0.15–0.25)	0.18 (0.16–0.20)	1.07 (0.81–1.42)	1.13 (0.85–1.49)
**Lymphoma/leukemia (C81-C96)**	1021 (2.11)	4448 (1.83)	3.50 (3.28–3.72)	2.64 (2.57–2.72)	1.30 (1.22–1.39) **	1.24 (1.16–1.33) **
**Solid metastasis (C77-C79)**	2618 (5.40)	12,202 (5.03)	8.97 (8.63–9.32)	7.27 (7.14–7.40)	1.25 (1.20–1.31) **	1.22 (1.17–1.27) **

Incident cancers in 48,493 patients with a diagnosis of visual impairment, including blindness (H54), compared to 242,465 population controls without visual impairment, matched according to birthyear, sex, and county. All patients were 40 years or older. Follow-up was censored for migration, death, and end of the study (31 December 2021), with controls also censored for H54 diagnosis. Event rates for all the outcomes were calculated as the number of events per 1000 person-years (pys) and are presented with exact Poisson 95% confidence intervals. Hazard ratios (HRs) with 95% confidence intervals (CIs) from Cox regression models comparing the H54 cases to the controls were calculated for all the outcomes, with adjustment for age, sex, and baseline year, as well as with adjustment for previous cancer (depending on the outcome). * *p* < 0.05, ** *p* < 0.001.

## Data Availability

Data cannot be made publicly available for ethical and legal reasons. Such information is subject to legal restrictions according to national legislation. Specifically, in Sweden, confidentiality regarding personal information in studies is regulated in the Public Access to Information and Secrecy Act (SFS 2009:400). The data underlying the results of this study might be made available upon request, after an assessment of confidentiality. Thus, there is a possibility to apply to get access to certain public documents that an authority holds. In this case, the University of Gothenburg is the specific authority that is responsible for the integrity of documents containing research data. Questions regarding such issues can be directed to the Head of the Institute of Medicine, Sahlgrenska Academy, University of Gothenburg, Gothenburg, Sweden. Contact information can be obtained from medicin@gu.se.

## Supplementary Materials

The following supporting information can be downloaded at: https://www.mdpi.com/article/10.3390/cancers18010147/s1, Table S1: Number of Visually Impaired (VI) per detailed diagnosis. Table S2: Incident Cancers in Visually Impaired (H54) vs. Population Controls—men only. Table S3: Incident Cancers in Visually Impaired (H54) vs. Population Controls—women only. Table S4: Incident Cancers in Visually Impaired (H54) vs. Population Controls—patients 40 to 64 years old. Table S5: Incident Cancers in Visually Impaired (H54) vs. Population Controls—patients 65 to 79 years old. Table S6: Incident Cancers in Visually Impaired (H54) vs. Population Controls—patients 80 years and older.

## Abbreviations

The following abbreviations are used in this manuscript:

VI	Visual impairment
HR	Hazard ratio
IQR	Inter-quartile range
CI	Confidence interval
LAN	Light at night
SES	Socioeconomic status

## References

[1] GBD 2019 Blindness and Vision Impairment Collaborators, Vision Loss Expert Group of the Global Burden of Disease Study (2021). Causes of blindness and vision impairment in 2020 and trends over 30 years, and prevalence of avoidable blindness in relation to VISION 2020: The Right to Sight: An analysis for the Global Burden of Disease Study. Lancet Glob. Health.

[2] Bourne R.R., Stevens G.A., White R.A., Smith J.L., Flaxman S.R., Price H., Jonas J.B., Keeffe J., Leasher J., Naidoo K. (2013). Causes of vision loss worldwide, 1990–2010: A systematic analysis. Lancet Glob. Health.

[3] Que L., Zhu Q., Jiang C., Lu Q. (2025). An analysis of the global, regional, and national burden of blindness and vision loss between 1990 and 2021: The findings of the Global Burden of Disease Study 2021. Front. Public Health.

[4] Tan T.E., Wong T.Y. (2022). Diabetic retinopathy: Looking forward to 2030. Front. Endocrinol..

[5] Assi L., Chamseddine F., Ibrahim P., Sabbagh H., Rosman L., Congdon N., Evans J., Ramke J., Kuper H., Burton M.J. (2021). A Global Assessment of Eye Health and Quality of Life: A Systematic Review of Systematic Reviews. JAMA Ophthalmol..

[6] Court H., McLean G., Guthrie B., Mercer S.W., Smith D.J. (2014). Visual impairment is associated with physical and mental comorbidities in older adults: A cross-sectional study. BMC Med..

[7] Hahn R.A. (1991). Profound bilateral blindness and the incidence of breast cancer. Epidemiology.

[8] Feychting M., Osterlund B., Ahlbom A. (1998). Reduced cancer incidence among the blind. Epidemiology.

[9] Pukkala E., Verkasalo P.K., Ojamo M., Rudanko S.L. (1999). Visual impairment and cancer: A population-based cohort study in Finland. Cancer Causes Control.

[10] Verkasalo P.K., Pukkala E., Stevens R.G., Ojamo M., Rudanko S.L. (1999). Inverse association between breast cancer incidence and degree of visual impairment in Finland. Br. J. Cancer.

[11] Kliukiene J., Tynes T., Andersen A. (2001). Risk of breast cancer among Norwegian women with visual impairment. Br. J. Cancer.

[12] Flynn-Evans E.E., Stevens R.G., Tabandeh H., Schernhammer E.S., Lockley S.W. (2009). Total visual blindness is protective against breast cancer. Cancer Causes Control.

[13] Pukkala E., Ojamo M., Rudanko S.L., Stevens R.G., Verkasalo P.K. (2006). Does incidence of breast cancer and prostate cancer decrease with increasing degree of visual impairment. Cancer Causes Control.

[14] von Elm E., Altman D.G., Egger M., Pocock S.J., Gøtzsche P.C., Vandenbroucke J.P. (2007). The Strengthening the Reporting of Observational Studies in Epidemiology (STROBE) statement: Guidelines for reporting observational studies. Bull. World Health Organ..

[15] Ludvigsson J.F., Andersson E., Ekbom A., Feychting M., Kim J.-L., Reuterwall C., Heurgren M., Olausson P.O. (2011). External review and validation of the Swedish national inpatient register. BMC Public Health.

[16] Everhov A.H., Frisell T., Osooli M., Brooke H.L., Carlsen H.K., Modig K., Marild K., Lindstrom J., Skoldin K., Heurgren M. (2025). Diagnostic accuracy in the Swedish national patient register: A review including diagnoses in the outpatient register. Eur. J. Epidemiol..

[17] Charlson M.E., Pompei P., Ales K.L., MacKenzie C.R. (1987). A new method of classifying prognostic comorbidity in longitudinal studies: Development and validation. J. Chronic Dis..

[18] Prathap R., Kirubha S., Rajan A.T., Manoharan S., Elumalai K. (2024). The increasing prevalence of cancer in the elderly: An investigation of epidemiological trends. Aging Med..

[19] Rahib L., Wehner M.R., Matrisian L.M., Nead K.T. (2021). Estimated Projection of US Cancer Incidence and Death to 2040. JAMA Netw. Open.

[20] Chang W.C., Hsieh T.C., Hsu W.L., Chang F.L., Tsai H.R., He M.S. (2024). Diabetes and further risk of cancer: A nationwide population-based study. BMC Med..

[21] Garcia D.M. (2025). Retinal physiology in metabolic syndrome. Adv. Genet..

[22] Park B.R., Kim S.Y., Shin D.W., Yang H.K., Park J.H. (2017). Influence of Socioeconomic Status, Comorbidity, and Disability on Late-stage Cancer Diagnosis. Osong Public Health Res. Perspect..

[23] Dermarkarian C.R., Kini A.T., Al Othman B.A., Lee A.G. (2020). Neuro-Ophthalmic Manifestations of Intracranial Malignancies. J. Neuroophthalmol..

[24] Quigley C., Tong J.Y., Zhang A.S., Psaltis A.J., Selva D. (2025). Clinico-radiological features of optic nerve sheath schwannoma: Review and illustrative case. Eur. J. Ophthalmol..

[25] Mehlan J., Schuttauf F., Salamon J.M., Kordes U., Friedrich R.E., Mautner V.F. (2019). Manifestations and Treatment of Adult-onset Symptomatic Optic Pathway Glioma in Neurofibromatosis Type 1. Anticancer Res..

[26] Guymer R.H., Campbell T.G. (2023). Age-related macular degeneration. Lancet.

[27] Nguyen A.M., Arora K.S., Swenor B.K., Friedman D.S., Ramulu P.Y. (2015). Physical activity restriction in age-related eye disease: A cross-sectional study exploring fear of falling as a potential mediator. BMC Geriatr..

[28] Starkoff B.E., Lenz E.K., Lieberman L.J., Foley J., Too D. (2017). Physical activity patterns of adults with visual impairments. Br. J. Vis. Impair..

[29] Ross S.M., Haegele J.A., Abrahamson K., Schram B.M., Healy S. (2022). US adults with visual impairments meeting 24-h movement guidelines: Updated national prevalence estimates. Disabil. Health J..

[30] Hughes R.B., Robinson-Whelen S., Knudson C. (2022). Cancer Disparities Experienced by People with Disabilities. Int. J. Environ. Res. Public Health.

[31] Buja A., Lago L., Lago S., Vinelli A., Zanardo C., Baldo V. (2018). Marital status and stage of cancer at diagnosis: A systematic review. Eur. J. Cancer Care.

[32] Barrero J.A., Mockus I. (2022). Early menarche in visually impaired girls: Evidence and hypothesis of light-dark cycle disruption and blindness effect on puberty onset. Chronobiol. Int..

[33] Barsegian A., Kotlyar B., Lee J., Salifu M.O., McFarlane S.I. (2017). Diabetic Retinopathy: Focus on Minority Populations. Int. J. Clin. Endocrinol. Metab..

[34] Dai W.W., Gao J.M., He P., Ma Z., Tian X.X., Zheng X.Y. (2019). The association between socioeconomic status and visual disability among older adults in China. Int. J. Ophthalmol..

[35] Kousiouris P., Klavdianou O., Douglas K.A.A., Gouliopoulos N., Chatzistefanou K., Kantzanou M., Dimtsas G.S., Moschos M.M. (2022). Role of Socioeconomic Status (SES) in Globe Injuries: A Review. Clin. Ophthalmol..

[36] Nesemann J.M., Morocho-Alburqueque N., Quincho-Lopez A., Muñoz M., Liliana-Talero S., Harding-Esch E.M., Saboyá-Díaz M.I., Honorio-Morales H.A., Durand S., Carey-Angeles C.A. (2023). Association of vision impairment and blindness with socioeconomic status in adults 50 years and older from Alto Amazonas, Peru. Eye.

[37] Iverson E., Sukhai M., Quinn M.P., Aubin M.-J., Freeman E.E. (2025). Visual impairment, employment status, and reduction in income: The Canadian Longitudinal Study on Aging. Can. J. Ophthalmol..

[38] Brunes A., Heir T. (2022). Visual impairment and employment in Norway. BMC Public Health.

[39] Wu A.M., Morse A.R., Seiple W.H., Talwar N., Hansen S.O., Lee P.P., Stein J.D. (2021). Reduced Mammography Screening for Breast Cancer among Women with Visual Impairment. Ophthalmology.

[40] Ricciardi G.E., Cuciniello R., De Ponti E., Lunetti C., Pennisi F., Signorelli C., Renzi C. (2024). Disability and Participation in Colorectal Cancer Screening: A Systematic Review and Meta-Analysis. Curr. Oncol..

[41] Spencer C., Frick K., Gower E.W., Kempen J.H., Wolff J.L. (2009). Disparities in access to medical care for individuals with vision impairment. Ophthalmic Epidemiol..

[42] Stevens R.G., Davis S. (1996). The melatonin hypothesis: Electric power and breast cancer. Environ. Health Perspect..

[43] Stevens R.G. (1987). Electric power use and breast cancer: A hypothesis. Am. J. Epidemiol..

[44] Czeisler C.A., Shanahan T.L., Klerman E.B., Martens H., Brotman D.J., Emens J.S., Klein T., Rizzo J.F. (1995). Suppression of melatonin secretion in some blind patients by exposure to bright light. N. Engl. J. Med..

[45] Hull J.T., Czeisler C.A., Lockley S.W. (2018). Suppression of Melatonin Secretion in Totally Visually Blind People by Ocular Exposure to White Light: Clinical Characteristics. Ophthalmology.

[46] Brown A.S., Best M.R., Mitchell D.B. (2013). More than meets the eye: Implicit perception in legally blind individuals. Conscious. Cogn..

[47] Phillips A.J.K., Vidafar P., Burns A.C., McGlashan E.M., Anderson C., Rajaratnam S.M.W., Lockley S.W., Cain S.W. (2019). High sensitivity and interindividual variability in the response of the human circadian system to evening light. Proc. Natl. Acad. Sci. USA.

[48] Chellappa S.L. (2021). Individual differences in light sensitivity affect sleep and circadian rhythms. Sleep.

[49] Spitschan M., Santhi N. (2022). Individual differences and diversity in human physiological responses to light. EBioMedicine.

[50] Choi S., Nanda P., Yuen K., Ong K. (2023). Bridging the gap in health literacy research: The inclusion of individuals with visual impairments. Patient Educ. Couns..

