# Supplementary material for: Visual Impairment and Cancer Risk: A Nationwide Cohort Study of Adult Swedish Men and Women
Source: Cancers (Basel). 2025 Dec 31;18(1):147. doi: 10.3390/cancers18010147 (PMC12784979; doi:10.3390/cancers18010147)
Supplement: Supplementary file 1 [file cancers-18-00147-s001.zip › cancers-4019291-supplementary.pdf]

Supporting information

to

Visual impairment and cancer risk: a nationwide cohort study of adult Swedish men and women

By

Leda Pistiolis, Henrik Litsne, Roger Olofsson Bagge,
Kristian F. Axelsson.

Table of contents

Table S1. Number of Visually Impaired (VI) per detailed diagnosis.

Table S2. Incident Cancers in Visually Impaired (H54) vs Population Controls – men only.

Table S3. Incident Cancers in Visually Impaired (H54) vs Population Controls – women only.

Table S4. Incident Cancers in Visually Impaired (H54) vs Population Controls – patients 40 to 64 years old.

Table S5. Incident Cancers in Visually Impaired (H54) vs Population Controls – patients 65 to 79 years old.

Table S6. Incident Cancers in Visually Impaired (H54) vs Population Controls – patients 80 years and older.

Table S1. Number of Visually Impaired (VI) per detailed diagnosis

**Case patients**	**N=**
H540 blind both eyes	3,654
H541 severe VI both eyes	4,517
H542 moderate VI both eyes	5,811
H543 mild VI both eyes	1,735
H544 blind one eye	12,748
H545 severe VI one eye	6,553
H546 moderate VI one eye	3,941
H547 mild VI one eye	3,363
H549 unspecified VI both eyes	6,171

Table S2. Incident Cancers in Visually Impaired (H54) vs Population Controls – men only

	n (%)		Incident rate per 1000 pys		HR (95%CI)	
**Incident Cancer Event**	**H54**	**Ctrls**	**H54**	**Ctrls**	**Adj. age, sex, year**	**+ adj. for prev cancer**
**Any cancer (C00-C97)**	5,798 (27.2)	29,128 (27.3)	53.1 (51.7-54.4)	45.4 (44.9-46.0)	1.18 (1.14-1.21)**	1.17 (1.13-1.20)**
**Oral neoplasms (C00-C14)**	140 (0.66)	504 (0.47)	1.09 (0.92-1.29)	0.67 (0.62-0.74)	1.60 (1.32-1.93)**	1.48 (1.22-1.78)**
**Esophagus (C15)**	68 (0.32)	330 (0.31)	0.53 (0.41-0.67)	0.44 (0.39-0.49)	1.23 (0.95-1.60)	1.09 (0.84-1.42)
**Stomach (C16)**	97 (0.45)	477 (0.45)	0.76 (0.61-0.92)	0.64 (0.58-0.70)	1.22 (0.98-1.51)	1.21 (0.97-1.51)
**Small intestine (C17)**	35 (0.16)	135 (0.13)	0.27 (0.19-0.38)	0.18 (0.15-0.21)	1.51 (1.04-2.19)*	1.52 (1.05-2.21)*
**Colon (C18)**	358 (1.68)	1,762 (1.65)	2.81 (2.52-3.11)	2.37 (2.26-2.48)	1.21 (1.08-1.36)**	1.19 (1.06-1.33)*
**Rectum (C20)**	186 (0.87)	911 (0.85)	1.45 (1.25-1.68)	1.22 (1.14-1.30)	1.20 (1.02-1.40)*	1.13 (0.97-1.33)
**Liver (C22)**	91 (0.43)	402 (0.38)	0.71 (0.57-0.87)	0.54 (0.49-0.59)	1.35 (1.08-1.70)*	1.30 (1.03-1.63)*
**Pancreas (C25)**	145 (0.68)	653 (0.61)	1.13 (0.95-1.33)	0.87 (0.81-0.94)	1.33 (1.11-1.59)*	1.34 (1.12-1.61)*
**Lung (C34)**	386 (1.81)	1,595 (1.50)	3.02 (2.72-3.33)	2.13 (2.03-2.24)	1.44 (1.28-1.60)**	1.47 (1.31-1.64)**
**Melanoma (C43)**	235 (1.10)	1,341 (1.26)	1.84 (1.61-2.09)	1.80 (1.71-1.90)	1.05 (0.91-1.20)	1.03 (0.89-1.18)
**Skin (C44)**	1,539 (7.21)	9,371 (8.78)	12.6 (11.9-13.2)	13.2 (12.9-13.4)	0.99 (0.94-1.04)	0.99 (0.94-1.04)
**Breast (C50)**	19 (0.09)	67 (0.06)	0.15 (0.09-0.23)	0.09 (0.07-0.11)	1.66 (1.00-2.76)*	1.58 (0.95-2.63)
**Prostate (C61)**	2,060 (9.66)	10,983 (10.3)	17.0 (16.3-17.8)	15.6 (15.3-15.9)	1.09 (1.04-1.14)**	1.11 (1.06-1.17)**
**Urinary bladder (C67)**	498 (2.33)	2,511 (2.35)	3.93 (3.59-4.29)	3.39 (3.26-3.53)	1.15 (1.05-1.27)*	1.17 (1.06-1.29)*
**Brain (C71)**	127 (0.60)	267 (0.25)	0.99 (0.83-1.18)	0.36 (0.31-0.40)	2.68 (2.17-3.31)**	1.86 (1.48-2.33)**
**Thyroid (C73)**	16 (0.07)	97 (0.09)	0.12 (0.07-0.20)	0.13 (0.10-0.16)	0.93 (0.55-1.58)	0.88 (0.52-1.50)
**Lymphoma/leukaemia (C81-C96)**	537 (2.52)	2,417 (2.27)	4.24 (3.89-4.61)	3.26 (3.13-3.39)	1.28 (1.16-1.40)**	1.18 (1.07-1.29)**
**Solid metastasis (C77-C79)**	1,294 (6.07)	6,357 (5.96)	10.2 (9.64-10.8)	8.57 (8.36-8.79)	1.22 (1.15-1.30)**	1.18 (1.11-1.25)**

Incident cancers in 21,335 male patients with diagnosis of visually impairment including blindness (H54) compared to 106,675 population controls without, matched according to birthyear, sex and county, all patients 40 years or older. Follow-up censored for migration, death and end of study (2021-12-31), with controls also censored for H54 diagnosis. Event rates for all the outcomes were calculated as the number of events per 1000 person-years (pys) and are presented with exact Poisson 95% confidence intervals. Hazard ratios (HR) with 95% confidence intervals (CI) from Cox regression models comparing the H54 cases to the controls were calculated for all the outcomes with adjustment for age, sex and baseline year as well as with added adjustment for previous cancer (depending on outcome). *p<0.05, **p<0.001.

Table S3. Incident Cancers in Visually Impaired (H54) vs Population Controls – women only

Incident Cancer Event	n (%)		Incident rate per 1000 pys		HR (95%CI)	
	H54	Ctrls	H54	Ctrls	Adj. age, sex, year	+ adj. for prev cancer
**Any cancer (C00-C97)**	5,289 (19.5)	25,569 (18.8)	35.8 (34.8-36.8)	30.1 (29.7-30.5)	1.19 (1.15-1.22)**	1.17 (1.14-1.20)**
**Oral neoplasms (C00-C14)**	107 (0.39)	440 (0.32)	0.64 (0.53-0.78)	0.47 (0.42-0.51)	1.38 (1.11-1.70)*	1.32 (1.07-1.64)*
**Esophagus (C15)**	34 (0.13)	162 (0.12)	0.20 (0.14-0.28)	0.17 (0.15-0.20)	1.20 (0.83-1.74)	1.06 (0.73-1.54)
**Stomach (C16)**	76 (0.28)	283 (0.21)	0.46 (0.36-0.57)	0.30 (0.27-0.34)	1.54 (1.19-1.98)**	1.48 (1.15-1.91)*
**Small intestine (C17)**	40 (0.15)	134 (0.10)	0.24 (0.17-0.33)	0.14 (0.12-0.17)	1.66 (1.16-2.36)*	1.21 (0.84-1.75)
**Colon (C18)**	409 (1.51)	1,929 (1.42)	2.47 (2.23-2.72)	2.05 (1.96-2.14)	1.23 (1.10-1.36)**	1.21 (1.09-1.34)**
**Rectum (C20)**	120 (0.44)	667 (0.49)	0.72 (0.60-0.86)	0.71 (0.65-0.76)	1.02 (0.84-1.24)	1.05 (0.86-1.27)
**Liver (C22)**	61 (0.22)	273 (0.20)	0.37 (0.28-0.47)	0.29 (0.26-0.32)	1.29 (0.98-1.70)	1.27 (0.96-1.67)
**Pancreas (C25)**	135 (0.50)	814 (0.60)	0.81 (0.68-0.96)	0.86 (0.80-0.92)	0.96 (0.80-1.16)	0.94 (0.78-1.12)
**Lung (C34)**	396 (1.46)	1,590 (1.17)	2.38 (2.15-2.63)	1.68 (1.60-1.77)	1.41 (1.27-1.58)**	1.28 (1.15-1.43)**
**Melanoma (C43)**	211 (0.78)	1,136 (0.84)	1.27 (1.10-1.45)	1.20 (1.14-1.28)	1.06 (0.92-1.23)	1.03 (0.89-1.20)
**Skin (C44)**	1,671 (6.15)	9,932 (7.31)	10.4 (9.95-11.0)	11.0 (10.8-11.2)	0.97 (0.92-1.02)	0.96 (0.91-1.01)
**Breast (C50)**	977 (3.60)	4,625 (3.41)	6.01 (5.64-6.40)	4.99 (4.85-5.14)	1.18 (1.10-1.27)**	1.18 (1.10-1.26)**
**Uterus (C54)**	253 (0.93)	1,110 (0.82)	1.53 (1.34-1.73)	1.18 (1.11-1.25)	1.27 (1.11-1.46)**	1.21 (1.06-1.39)*
**Ovary (C56)**	123 (0.45)	601 (0.44)	0.74 (0.61-0.88)	0.64 (0.59-0.69)	1.14 (0.94-1.38)	1.12 (0.92-1.36)
**Urinary bladder (C67)**	203 (0.75)	861 (0.63)	1.22 (1.06-1.40)	0.91 (0.85-0.97)	1.34 (1.15-1.56)**	1.35 (1.16-1.57)**
**Brain (C71)**	97 (0.36)	217 (0.16)	0.58 (0.47-0.71)	0.23 (0.20-0.26)	2.47 (1.94-3.14)**	1.95 (1.52-2.51)**
**Thyroid (C73)**	42 (0.15)	201 (0.15)	0.25 (0.18-0.34)	0.21 (0.18-0.24)	1.14 (0.82-1.59)	1.23 (0.88-1.72)
**Lymphoma/leukaemia (C81-C96)**	484 (1.78)	2,031 (1.50)	2.93 (2.67-3.20)	2.16 (2.07-2.25)	1.33 (1.21-1.47)**	1.34 (1.21-1.48)**
**Solid metastasis (C77-C79)**	1,324 (4.88)	5,845 (4.30)	8.04 (7.61-8.48)	6.23 (6.08-6.40)	1.29 (1.22-1.37)**	1.26 (1.19-1.34)**

Incident cancers in 27,158 female patients with diagnosis of visually impairment including blindness (H54) compared to 135,790 population controls without, matched according to birthyear, sex and county, all patients 40 years or older. Follow-up censored for migration, death and end of study (2021-12-31), with controls also censored for H54 diagnosis. Event rates for all the outcomes were calculated as the number of events per 1000 person-years (pys) and are presented with exact Poisson 95% confidence intervals. Hazard ratios (HR) with 95% confidence intervals (CI) from Cox regression models comparing the H54 cases to the controls were calculated for all the outcomes with adjustment for age, sex and baseline year as well as with added adjustment for previous cancer (depending on outcome). *p<0.05, **p<0.001.

Table S4. Incident Cancers in Visually Impaired (H54) vs Population Controls – patients 40 to 64 years old

Incident Cancer Event	n (%)		Incident rate per 1000 pys		HR (95%CI)	
	H54	Ctrls	H54	Ctrls	Adj. age, sex, year	+ adj. for prev cancer
**Any cancer (C00-C97)**	2,168 (17.3)	8,847 (14.1)	20.8 (19.9-21.7)	15.4 (15.1-15.8)	1.37 (1.30-1.43)**	1.31 (1.25-1.38)**
**Oral neoplasms (C00-C14)**	76 (0.60)	237 (0.38)	0.67 (0.52-0.83)	0.38 (0.34-0.44)	1.74 (1.34-2.25)**	1.78 (1.37-2.31)**
**Esophagus (C15)**	22 (0.18)	83 (0.13)	0.19 (0.12-0.29)	0.13 (0.11-0.17)	1.49 (0.93-2.38)	1.16 (0.73-1.86)
**Stomach (C16)**	26 (0.21)	129 (0.21)	0.23 (0.15-0.33)	0.21 (0.17-0.25)	1.12 (0.74-1.71)	1.05 (0.69-1.61)
**Small intestine (C17)**	14 (0.11)	52 (0.08)	0.12 (0.07-0.21)	0.08 (0.06-0.11)	1.47 (0.82-2.66)	1.21 (0.66-2.22)
**Colon (C18)**	112 (0.89)	479 (0.76)	0.98 (0.81-1.18)	0.78 (0.71-0.85)	1.30 (1.05-1.59)*	1.31 (1.07-1.61)*
**Rectum (C20)**	54 (0.43)	297 (0.47)	0.47 (0.36-0.62)	0.48 (0.43-0.54)	1.01 (0.75-1.35)	0.96 (0.72-1.29)
**Liver (C22)**	42 (0.33)	120 (0.19)	0.37 (0.26-0.50)	0.19 (0.16-0.23)	1.95 (1.38-2.78)**	1.87 (1.31-2.66)**
**Pancreas (C25)**	53 (0.42)	231 (0.37)	0.46 (0.35-0.61)	0.37 (0.33-0.43)	1.28 (0.95-1.73)	1.20 (0.89-1.62)
**Lung (C34)**	186 (1.48)	574 (0.91)	1.63 (1.40-1.88)	0.93 (0.86-1.01)	1.80 (1.53-2.12)**	1.62 (1.37-1.91)**
**Melanoma (C43)**	97 (0.77)	528 (0.84)	0.85 (0.69-1.04)	0.86 (0.79-0.93)	1.01 (0.81-1.25)	1.02 (0.82-1.27)
**Skin (C44)**	512 (4.07)	2,560 (4.07)	4.58 (4.19-4.99)	4.24 (4.07-4.40)	1.10 (1.00-1.21)*	1.09 (0.99-1.20)
**Breast (C50)**	320 (2.55)	1,306 (2.08)	2.84 (2.54-3.17)	2.14 (2.03-2.26)	1.31 (1.16-1.48)**	1.25 (1.10-1.41)**
**Uterus (C54)**	49 (0.39)	232 (0.37)	0.43 (0.32-0.57)	0.38 (0.33-0.43)	1.14 (0.84-1.55)	1.10 (0.81-1.49)
**Ovary (C56)**	41 (0.33)	172 (0.27)	0.36 (0.26-0.49)	0.28 (0.24-0.32)	1.27 (0.90-1.79)	1.27 (0.90-1.79)
**Prostate (C61)**	249 (1.98)	1,402 (2.23)	2.20 (1.94-2.49)	2.30 (2.18-2.42)	1.01 (0.88-1.15)	1.02 (0.89-1.17)
**Urinary bladder (C67)**	81 (0.64)	336 (0.53)	0.71 (0.56-0.88)	0.54 (0.49-0.61)	1.35 (1.06-1.72)*	1.17 (0.91-1.49)
**Brain (C71)**	103 (0.82)	136 (0.22)	0.90 (0.74-1.09)	0.22 (0.18-0.26)	4.05 (3.13-5.23)**	2.69 (2.03-3.55)**
**Thyroid (C73)**	20 (0.16)	98 (0.16)	0.17 (0.11-0.27)	0.16 (0.13-0.19)	1.08 (0.67-1.75)	1.12 (0.69-1.82)
**Lymphoma/leukaemia (C81-C96)**	224 (1.78)	612 (0.97)	1.97 (1.72-2.25)	0.99 (0.92-1.08)	1.99 (1.71-2.32)**	1.74 (1.49-2.03)**
**Solid metastasis (C77-C79)**	572 (4.55)	2,150 (3.42)	5.05 (4.64-5.48)	3.51 (3.36-3.66)	1.47 (1.34-1.61)**	1.41 (1.29-1.55)**

Incident cancers in 12,565 patients 40 to 64 years old at baseline with diagnosis of visually impairment including blindness (H54) compared to 62,825 population controls without, matched according to birthyear, sex and county. Follow-up censored for migration, death and end of study (2021-12-31), with controls also censored for H54 diagnosis. Event rates for all the outcomes were calculated as the number of events per 1000 person-years (pys) and are presented with exact Poisson 95% confidence intervals. Hazard ratios (HR) with 95% confidence intervals (CI) from Cox regression models comparing the H54 cases to the controls were calculated for all the outcomes with adjustment for age, sex and baseline year as well as with added adjustment for previous cancer (depending on outcome). *p<0.05, **p<0.001.

Table S5. Incident Cancers in Visually Impaired (H54) vs Population Controls – patients 65 to 79 years old

Incident Cancer Event	n (%)		Incident rate per 1000 pys		HR (95%CI)	
	H54	Ctrls	H54	Ctrls	Adj. age, sex, year	+ adj. for prev cancer
**Any cancer (C00-C97)**	4,359 (28.9)	21,726 (28.8)	51.4 (49.9-53.0)	43.4 (42.8-44.0)	1.17 (1.13-1.20)**	1.16 (1.13-1.20)**
**Oral neoplasms (C00-C14)**	100 (0.66)	361 (0.48)	0.99 (0.81-1.20)	0.61 (0.55-0.68)	1.59 (1.27-1.98)**	1.44 (1.16-1.80)*
**Esophagus (C15)**	50 (0.33)	237 (0.31)	0.49 (0.37-0.65)	0.40 (0.35-0.45)	1.24 (0.91-1.68)	1.22 (0.90-1.66)
**Stomach (C16)**	75 (0.50)	328 (0.44)	0.74 (0.58-0.93)	0.55 (0.49-0.62)	1.34 (1.05-1.73)*	1.30 (1.01-1.67)*
**Small intestine (C17)**	40 (0.27)	124 (0.16)	0.40 (0.28-0.54)	0.21 (0.17-0.25)	1.85 (1.29-2.64)**	1.80 (1.26-2.58)*
**Colon (C18)**	315 (2.09)	1,590 (2.11)	3.14 (2.80-3.50)	2.70 (2.57-2.84)	1.16 (1.03-1.31)*	1.13 (1.00-1.28)*
**Rectum (C20)**	142 (0.94)	708 (0.94)	1.41 (1.19-1.66)	1.20 (1.11-1.29)	1.16 (0.97-1.39)	1.16 (0.97-1.39)
**Liver (C22)**	60 (0.40)	312 (0.41)	0.59 (0.45-0.76)	0.53 (0.47-0.59)	1.13 (0.85-1.48)	1.10 (0.84-1.45)
**Pancreas (C25)**	130 (0.86)	659 (0.87)	1.28 (1.07-1.52)	1.11 (1.03-1.20)	1.16 (0.96-1.40)	1.19 (0.98-1.43)
**Lung (C34)**	376 (2.49)	1,625 (2.16)	3.73 (3.36-4.13)	2.75 (2.62-2.89)	1.34 (1.20-1.50)**	1.28 (1.15-1.44)**
**Melanoma (C43)**	185 (1.23)	1,021 (1.35)	1.84 (1.58-2.12)	1.73 (1.63-1.84)	1.07 (0.91-1.25)	1.07 (0.91-1.25)
**Skin (C44)**	1,186 (7.87)	7,336 (9.73)	12.4 (11.7-13.1)	13.1 (12.8-13.4)	0.95 (0.89-1.01)	0.94 (0.89-1.00)
**Breast (C50)**	373 (2.47)	1,656 (2.20)	3.74 (3.37-4.14)	2.83 (2.69-2.97)	1.27 (1.13-1.42)**	1.28 (1.15-1.44)**
**Uterus (C54)**	110 (0.73)	475 (0.63)	1.09 (0.90-1.31)	0.80 (0.73-0.88)	1.29 (1.05-1.59)*	1.23 (1.00-1.52)*
**Ovary (C56)**	46 (0.31)	267 (0.35)	0.45 (0.33-0.61)	0.45 (0.40-0.51)	0.96 (0.70-1.32)	0.97 (0.71-1.33)
**Prostate (C61)**	837 (5.55)	4,634 (6.15)	8.59 (8.02-9.20)	8.11 (7.88-8.35)	1.04 (0.96-1.12)	1.08 (1.00-1.16)*
**Urinary bladder (C67)**	280 (1.86)	1,452 (1.93)	2.80 (2.48-3.14)	2.47 (2.35-2.60)	1.11 (0.98-1.26)	1.10 (0.97-1.25)
**Brain (C71)**	90 (0.60)	205 (0.27)	0.89 (0.72-1.09)	0.35 (0.30-0.40)	2.47 (1.93-3.17)**	1.96 (1.51-2.53)**
**Thyroid (C73)**	24 (0.16)	109 (0.14)	0.24 (0.15-0.35)	0.18 (0.15-0.22)	1.23 (0.79-1.92)	1.29 (0.82-2.01)
**Lymphoma/leukaemia (C81-C96)**	401 (2.66)	1,869 (2.48)	4.01 (3.63-4.43)	3.18 (3.04-3.33)	1.22 (1.10-1.36)**	1.17 (1.05-1.31)*
**Solid metastasis (C77-C79)**	1,177 (7.81)	5,470 (7.26)	11.8 (11.1-12.5)	9.35 (9.10-9.60)	1.26 (1.18-1.34)**	1.21 (1.14-1.29)**

Incident cancers in 15,074 patients 65 to 79 years old at baseline with diagnosis of visually impairment including blindness (H54) compared to 75,370 population controls without, matched according to birthyear, sex and county. Follow-up censored for migration, death and end of study (2021-12-31), with controls also censored for H54 diagnosis. Event rates for all the outcomes were calculated as the number of events per 1000 person-years (pys) and are presented with exact Poisson 95% confidence intervals. Hazard ratios (HR) with 95% confidence intervals (CI) from Cox regression models comparing the H54 cases to the controls were calculated for all the outcomes with adjustment for age, sex and baseline year as well as with added adjustment for previous cancer (depending on outcome). *p<0.05, **p<0.001.

Table S6. Incident Cancers in Visually Impaired (H54) vs Population Controls – patients 80 years and older

Incident Cancer Event	n (%)		Incident rate per 1000 pys		HR (95%CI)	
	H54	Ctrls	H54	Ctrls	Adj. age, sex, year	+ adj. for prev cancer
**Any cancer (C00-C97)**	4,560 (21.9)	24,124 (23.1)	66.9 (65.0-68.9)	58.0 (57.2-58.7)	1.11 (1.08-1.15)**	1.10 (1.07-1.14)**
**Oral neoplasms (C00-C14)**	71 (0.34)	346 (0.33)	0.89 (0.70-1.13)	0.71 (0.64-0.79)	1.22 (0.94-1.58)	1.14 (0.88-1.47)
**Esophagus (C15)**	30 (0.14)	172 (0.16)	0.38 (0.25-0.54)	0.35 (0.30-0.41)	1.04 (0.70-1.53)	0.89 (0.60-1.31)
**Stomach (C16)**	72 (0.35)	303 (0.29)	0.91 (0.71-1.14)	0.62 (0.56-0.70)	1.41 (1.09-1.83)*	1.46 (1.13-1.89)*
**Small intestine (C17)**	21 (0.10)	93 (0.09)	0.26 (0.16-0.40)	0.19 (0.15-0.23)	1.28 (0.80-2.06)	1.11 (0.69-1.79)
**Colon (C18)**	340 (1.63)	1,622 (1.56)	4.31 (3.87-4.80)	3.37 (3.21-3.53)	1.24 (1.10-1.39)**	1.22 (1.08-1.37)**
**Rectum (C20)**	110 (0.53)	573 (0.55)	1.39 (1.14-1.67)	1.18 (1.09-1.29)	1.12 (0.91-1.38)	1.06 (0.86-1.29)
**Liver (C22)**	50 (0.24)	243 (0.23)	0.63 (0.47-0.83)	0.50 (0.44-0.57)	1.24 (0.92-1.69)	1.24 (0.92-1.69)
**Pancreas (C25)**	97 (0.47)	577 (0.55)	1.22 (0.99-1.49)	1.19 (1.09-1.29)	1.00 (0.81-1.24)	0.99 (0.80-1.22)
**Lung (C34)**	220 (1.05)	986 (0.95)	2.77 (2.42-3.16)	2.04 (1.91-2.17)	1.30 (1.13-1.51)**	1.32 (1.14-1.53)**
**Melanoma (C43)**	164 (0.79)	928 (0.89)	2.07 (1.77-2.41)	1.92 (1.80-2.05)	1.06 (0.90-1.25)	0.99 (0.84-1.17)
**Skin (C44)**	1,512 (7.25)	9,407 (9.02)	20.2 (19.2-21.3)	20.8 (20.4-21.3)	0.96 (0.91-1.01)	0.95 (0.90-1.01)
**Breast (C50)**	303 (1.45)	1,730 (1.66)	3.85 (3.43-4.31)	3.60 (3.44-3.78)	1.00 (0.89-1.13)	1.01 (0.90-1.14)
**Uterus (C54)**	94 (0.45)	408 (0.39)	1.18 (0.96-1.45)	0.84 (0.76-0.93)	1.30 (1.04-1.63)*	1.20 (0.96-1.50)
**Ovary (C56)**	36 (0.17)	162 (0.16)	0.45 (0.32-0.63)	0.33 (0.28-0.39)	1.25 (0.87-1.79)	1.17 (0.82-1.68)
**Prostate (C61)**	974 (4.67)	4,954 (4.75)	12.6 (11.8-13.4)	10.5 (10.2-10.8)	1.15 (1.07-1.23)**	1.16 (1.09-1.25)**
**Urinary bladder (C67)**	340 (1.63)	1,584 (1.52)	4.32 (3.87-4.80)	3.30 (3.14-3.46)	1.24 (1.10-1.39)**	1.30 (1.15-1.46)**
**Brain (C71)**	31 (0.15)	143 (0.14)	0.39 (0.26-0.55)	0.29 (0.25-0.35)	1.30 (0.88-1.91)	1.12 (0.75-1.66)
**Thyroid (C73)**	14 (0.07)	91 (0.09)	0.18 (0.10-0.30)	0.19 (0.15-0.23)	0.86 (0.49-1.52)	0.83 (0.47-1.46)
**Lymphoma/leukaemia (C81-C96)**	396 (1.90)	1,967 (1.89)	5.03 (4.55-5.55)	4.09 (3.91-4.28)	1.15 (1.03-1.28)*	1.11 (1.00-1.24)
**Solid metastasis (C77-C79)**	869 (4.17)	4,582 (4.39)	11.0 (10.3-11.8)	9.53 (9.26-9.81)	1.13 (1.05-1.22)**	1.12 (1.04-1.20)*

Incident cancers in 20,854 patients 80 years and older at baseline with diagnosis of visually impairment including blindness (H54) compared to 104,270 population controls without, matched according to birthyear, sex and county. Follow-up censored for migration, death and end of study (2021-12-31), with controls also censored for H54 diagnosis. Event rates for all the outcomes were calculated as the number of events per 1000 person-years (pys) and are presented with exact Poisson 95% confidence intervals. Hazard ratios (HR) with 95% confidence intervals (CI) from Cox regression models comparing the H54 cases to the controls were calculated for all the outcomes with adjustment for age, sex and baseline year as well as with added adjustment for previous cancer (depending on outcome). *p<0.05, **p<0.001.
